# A Case of Relapsing Kikuchi-Fujimoto Disease

**DOI:** 10.1155/2013/364795

**Published:** 2013-01-20

**Authors:** Talayeh Rezayat, Matthew B. Carroll, Bryan C. Ramsey, Andria Smith

**Affiliations:** Department of Internal Medicine, Keesler Medical Center, 301 Fisher Avenue, Biloxi, MS 39534, USA

## Abstract

Kikuchi-Fujimoto disease (KFD) or histiocytic necrotizing lymphadenitis was first described in Japan in 1972. It is described as a benign syndrome most commonly involving cervical lymphadenopathy, fever, and night sweats. The etiology of KFD is unknown but it is thought to be triggered by an autoimmune or viral process with an exaggerated T-cell-mediated immune response. KFD can mimic other serious conditions such as lymphoma, systemic lupus erythematosus (SLE), herpes simplex, and Epstein Barr virus. Diagnosis is confirmed histopathologically. Kikuchi's disease is typically reported to have a self-limiting course, resolving within several months and with a low recurrence rate between 3% and 4%. There is no specific treatment for KFD but any treatment is generally directed towards symptomatic relief with antipyretics and anti-inflammatory medications. In severe cases corticosteroids have been used. Here we describe a case of a previously healthy 26-year-old female that presented with fever and cervical lymphadenopathy. Malignancy and infections were ruled, and she was diagnosed with KFD histopathologically by lymph node biopsy. Her case is a severe case of KFD that despite treatment with multiple courses of corticosteroids and an immune modulating agent, relapsed.

## 1. Introduction

Kikuchi-Fujimoto disease (KFD) or histiocytic necrotizing lymphadenitis was first described in Japan in 1972 [1, 2]. It has been described as a benign syndrome most commonly involving cervical lymphadenopathy, fever, and night sweats. The etiology of KFD is unknown but it is thought to be triggered by an autoimmune or viral processwith an exaggerated T-cell-mediated immune response. KFD can be mistaken with other serious conditions such as lymphoma, systemic lupus erythematosus (SLE), herpes simplex, and Epstein Barr virus, to name a few. Diagnosis is confirmed histopathologically. Kikuchi's disease is typically reported to have a self-limiting course, resolving within several months and with a low recurrence rate between 3% to 4% [3]. There is no specific treatment for KFD but any treatment is generally directed towards symptomatic relief with antipyretics and anti-inflammatory medications. In severe cases, corticosteroids have been used without relapse of disease. Here we present a severe case of KFD that recurred despite multiple doses of corticosteroids and an additional immune modulating agent.

## 2. Case Presentation

An otherwise healthy 26-year-old African American female presented with progressive fever, headache, facial edema, and periorbital swelling of 3 weeks duration. She also complained of nausea, decreased appetite, and back pain. She had daily fevers up to 104.1°F with associated tachycardia. On physical exam, she had significant facial and periorbital swelling, preauricular, and anterior cervical tender lymphadenopathy. There was no evidence of scleral icterus or hepatosplenomegaly. Her complete blood count revealed a pancytopenia with a WBC count of 2,000 cells/mm^3^ and the presence of 1% atypical lymphocytes with an increase in immature neutrophils (“bands”), a hemoglobin of 11.3 g/dL, and a mild thrombocytopenia. Additional testing revealed elevated transaminases. An erythrocyte sedimentation rate (ESR) at time of her initial presentation was minimally elevated. C-reactive protein (CRP), antinuclear antibody (ANA), and angiotensin converting enzyme (ACE) were all normal, as shown in Table 1.

**Figure 1:**
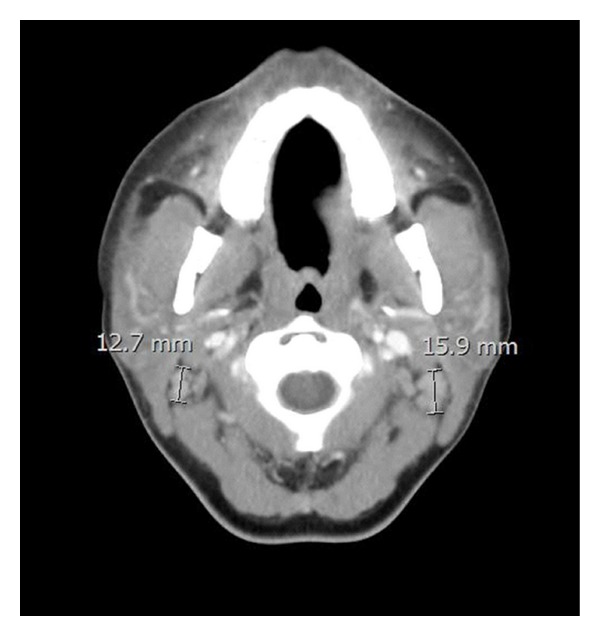
Cervical lymphadenopathy associated with Kikuchi-Fujimoto's Disease.

Computer tomography (CT) imaging of her neck was performed and revealed abnormally enhancing cervical and supraclavicular lymphadenopathy with diffuse salivary and lacrimal gland enlargement (Figure 1). Infectious etiologies such as mononucleosis, human immunodeficiency virus (HIV), and other multiple viral, fungal, and bacterial serologies were tested for and were negative (Table 2).

With her history suspicious for lymphoma, an MR I of the brain was obtained that revealed posterior leptomeningeal enhancement concerning for possible meningitis or lymphoma. Cerebral spinal fluid obtained by lumbar puncture however did not demonstrate findings consistent with meningitis or neoplasia. An excisional cervical lymph node biopsy and bone marrow aspiration and biopsy were performed. The bone marrow biopsy revealed a slightly hypocellular marrow for age with trilineage hematopoiesis and less than 1% blasts. There were increased marrow histiocytes with focal evidence of hemophagocytosis and lymphohistiophagocytosis. The flow cytometry of the bone marrow was negative for lymphoma or leukemia and revealed a lymphoid population of approximately 14% of the nucleated/nonerythroid cells, of which 83% were T cells and 8% were polyclonal B cells. Despite the information provided by the bone marrow biopsy, a specific diagnosis was not reached. 

**Figure 2:**
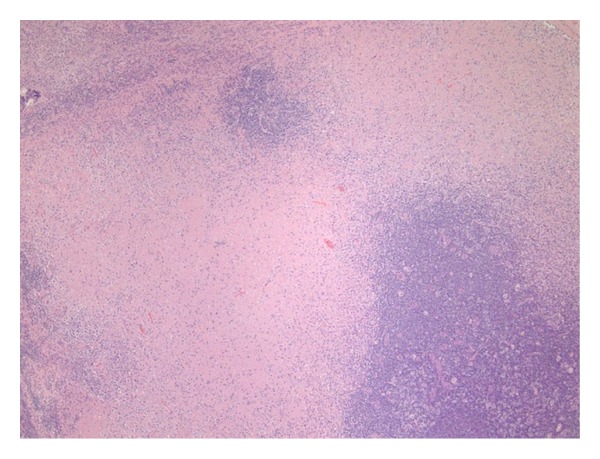
Cervical Lymph node disrupted architecture on Hematoxylin and Eosin (H&E) stain at 40X magnification.

**Figure 3:**
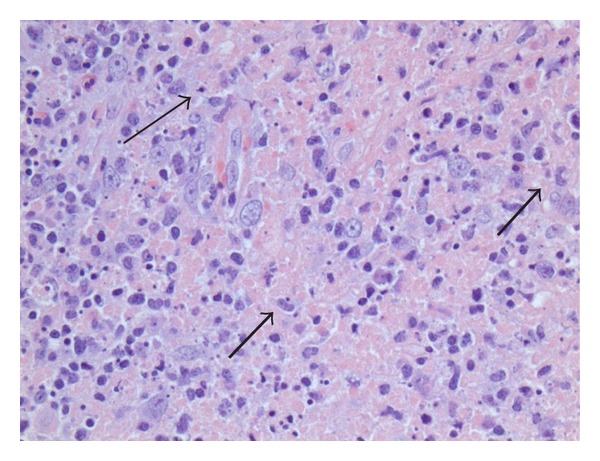
400X magnification of cervical lymph node biopsy showing disrupted architecture, paracortical expansion with areas of necrosis and nuclear debris (thin arrow). Plasma cells and numerous histiocytes (thick arrows) present. Above findings are consistent with KFD.

Excisional cervical lymph node biopsy was performed with results that confirmed a diagnosis of Kikuchi-Fujimoto's disease. The hematoxylin and eosin staining showed disrupted architecture with paracortical expansion, multifocal areas of necrosis, and cellular debris (Figure 2). Neutrophils were not prominent in the tissue obtained via lymph node biopsy but plasma cells and numerous histiocytes were identified (Figure 3). Immunohistochemical staining showed a predominance of CD3 positive T cells with a mixture of CD4 and CD8 positive cells.

This patient was started on prednisone 60 mg daily with rapid improvement in her lymphadenopathy, fevers, and other constitutional symptoms. She continued high dose corticosteroids with a slow taper over several months; however, after four months and reaching a dose of 5 mg a day of prednisone her symptoms relapsed necessitating reinitiation of high dose prednisone. Repeat serologic screening for systemic lupus erythematosus with an ANA was negative. Although symptoms improved with resuming a high dose of prednisone, her course was complicated by development of glucocorticoid-induced osteoporosis. Subsequently, she was started on hydroxychloroquine with a rapid steroid taper. She continues to be on hydroxychloroquine for symptom control but also experienced a relapse of her symptoms while on this medication and is currently being considered for dual immunosuppressive therapy to control her condition.

## 3. Discussion

Kikuchi-Fujimoto disease (KFD) is most commonly seen in adults younger than 40 years of age. A female predominance has been reported previously but recent reports reveal a ratio closer to 1 : 1 for males and females [2]. It has the highest prevalence in patients of Asian descent. The onset of KFD can be acute or subacute developing over several weeks. The majority of patients (56% to 98%) present with cervical lymphadenopathy. The next most common clinical manifestation is fever which occurs in 30% to 50% of affected patients [2]. Other less commonly reported findings included leukopenia (up to 50%), atypical lymphocytes on peripheral smear, liver dysfunction, and bone marrow involvement [1, 2]. Some viral infections such as Ebstein-Barr virus or human herpes virus 6 have been suggested as triggers for the onset of KFD but this hypothesis has not been confirmed. There have been case reports of patients concurrently developing the hemophagocytic syndrome and these two syndromes may be part of a continuum rather than two separate entities [2].

The diagnosis of KFD is made histopathologically typically from tissue obtained by excisional lymph node biopsy. Histopathological findings for KFD include irregular paracortical areas of coagulative necrosis with extensivekaryorrhectic debris and altered lymph node architecture [1–3]. There is an abundance of histiocytes and plasmacytoid monocytes with a relative absence of neutrophils. The majority of the lymphocyte population are T cells with few B cells present. Of those T cells, there is CD8+ predominance.

Clinically KFD can mimic lymphoma. Also to consider in the differential diagnosis are infectious etiologies such as mononucleosis, tuberculosis, herpes simplex virus, and SLE. As the treatment for each possible etiology is very different, correct diagnosis of KFD is important [1, 2].It has been hypothesized that individuals with KFD are more susceptible to SLE and should be routinely screened for this disorder [1, 4].

Most reports describe KFD as a benign self-limitedsyndrome with effective symptomatic treatment provided by anti-inflammatory and antipyretics. Glucocorticoids are typically used for symptomatic relief in severe cases [1, 4, 5]. Signs and symptoms related to KFD usually resolve after several months [1–5]. A low recurrence rate of 3% to 4% has been reported [1–3]. Recurrences have been treated with the same agents as the first occurrence. Rezai et al. reported the first case report of recurrent KFD treated with hydroxychloroquine lieu of using glucocorticoids [4].

Our patient did not have a benign, self-limited course. Her disease was complicated by immediate return of symptoms after 4 months of glucocorticoid therapy and before complete discontinuation. Furthermore, she experienced glucocorticoid-induced osteoporosis. Hydroxychloroquine was initiated as alternative to glucocorticoids because of its safer side effect profile. She continues to require treatment with hydroxychloroquine but as noted above has experienced a relapse of her disease on this agent and will likely need dual immunosuppressive therapy in the very near future.

## Figures and Tables

**Table 1:** Pertinent laboratory data at the time of the patient's initial evaluation.

	Admission value	Reference range
Albumin (g/dL)	3.6	3.5–5.2
Total protein (g/dL)	6.7	6.6–8.7
AST (U/L)	145	10–50
ALT (U/L)	74	10–50
Alkaline phosphatase (U/L)	54	40–130
Total bilirubin (mg/dL)	0.8	

WBC (per mcL)	2,000	4,500–11,000
Hemoglobin (g/dL)	11.3	12.0–15.0
Hematocrit (%)	34.0	36–48
Platelets (per mcL)	126,000	150,000–450,000

ESR (mm/hr)	23	0–20
CRP (mg/dL)	0.63	<0.80
ANA	Negative	Negative
ACE (U/L)	58	9–67

Key: AST: aspartate aminotransferase; ALT: alanine aminotransferase; WBC: white blood cell; ESR: erythrocyte sedimentation rate; CRP: C-reactive protein; ANA: antinuclear antibody; ACE: angiotensin converting enzyme.

**Table 2:** Pertinent infectious serologies performed during the patient's evaluation.

Serology	Result	Reference Range
Epstein-Barr Virus capsid AbIgG	Positive	Negative
Epstein-Barr Virus capsid AbIgM	Negative	Negative
Epstein-Barr Virus Nuclear AbIgG	Positive	Negative
Mononucleosis	Negative	Negative
Cytomegalovirus AbIgG	Positive	Negative
Cytomegalovirus AbIgM	Negative	Negative
Human immunodeficiency virus	Negative	Negative
Adenovirus Ab	1 : 16 Ab detected	<1 : 8 Ab not detected
Parvovirus B12 IgG	<0.1	<0.9 Negative
Parvovirus B12 IgM	0.1	<0.9 Negative
*Coxiellaburnetii* Ab	Negative	Negative
Hepatitis C Ab	Negative	Negative
Hepatitis A AbIgM	Negative	Negative
Hepatitis B surface Ag and core Ab	Negative	Negative
*Aspergillu sniger* Ab	Negative	Negative
*Aspergillus fumigatus* Ab	Negative	Negative
*Aspergillus flavus* Ab	Negative	Negative
*Blastomyces* species Ab	Negative	Negative
*Coccidioides immitis * Ab	Negative	Negative
*Histoplasma capsulate myeast* Ab	<1 : 8	<1 : 8 Negative
*Histoplasma capsulatum* mycelial Ab	<1 : 8	<1 : 8 Negative
*Toxoplasma gondii* AbIgG and IgM	Both negative	Negative
*Bartonella henselae* AbIgG and IgM	Both negative	Negative
*Bartonella quintana* AbIgG and IgM	Both negative	Negative

Key: Ab: antibody; Ag: antigen.
